# Research on a multi-dimensional image information fusion algorithm based on NSCT transform

**DOI:** 10.1007/s12200-023-00104-0

**Published:** 2024-01-23

**Authors:** Yuxiang Su, Xi Liang, Danhua Cao, Zhenyu Yang, Yuanlong Peng, Ming Zhao

**Affiliations:** 1 School of Optical and Electronic Information, Huazhong University of Science and Technology, Wuhan, 430074 China; 2 State Grid Information and Telecommunication Branch, Beijing, 100761 China

**Keywords:** Power inspection, Object detection, Polarization imaging, Image fusion, Image denoising

## Abstract

Traditional inspection cameras determine targets and detect defects by capturing images of their light intensity, but in complex environments, the accuracy of inspection may decrease. Information based on polarization of light can characterize various features of a material, such as the roughness, texture, and refractive index, thus improving classification and recognition of targets. This paper uses a method based on noise template threshold matching to denoise and preprocess polarized images. It also reports on design of an image fusion algorithm, based on NSCT transform, to fuse light intensity images and polarized images. The results show that the fused image improves both subjective and objective evaluation indicators, relative to the source image, and can better preserve edge information and help to improve the accuracy of target recognition. This study provides a reference for the comprehensive application of multi-dimensional optical information in power inspection.

**Graphical Abstract:**

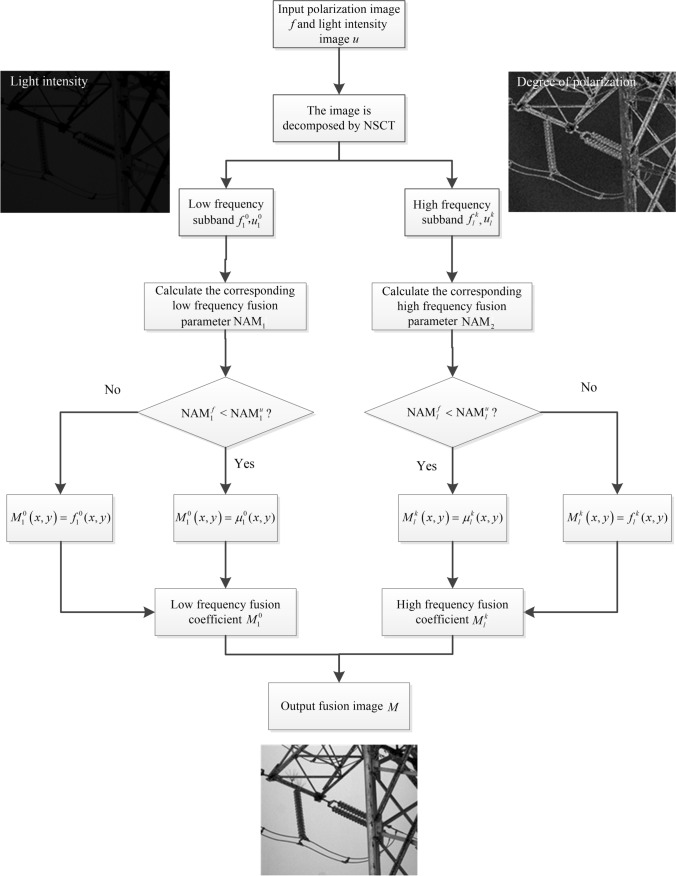

## Introduction

In the power grid industry, the video surveillance system is essential for intelligent inspection, encompassing three critical stages: transmission, distribution, and transformation. However, the complex monitoring environment and widely dispersed installations present challenges for acquiring high-quality images. Current industrial video surveillance relies on visible light-intensity or infrared light-intensity image information captured by traditional imaging chips, but the exclusive use of intensity image is insufficient to meet the recognition and detection requirements of the target.

Polarization is a fundamental property of light. Polarization imaging capture information about an object's refractive index, roughness, and other physical characteristics that remain unchanged regardless of external environmental conditions. This enables polarization imaging to have all-weather target detection and recognition capabilities [1]. It can successfully identify camouflaged targets and under hazy conditions as it has a longer range of object distance than traditional light-intensity imaging [2–4]. Additionally, polarization images can be used for target classification, soil water content detection, and other applications due to the physical information they carry about objects [5–7].

The intensity image is consistent with human vision, but sometimes the target cannot be completely distinguished from the background. Polarization image can distinguish the target more effectively and highlight the contour and texture details, although it does not conform to human visual perception. By employing image fusion techniques, these two types of images can be combined to effectively reveal multi-dimensional features. This fusion process compensates for the limitations of information from a single image sensor, providing more reliable and accurate target information [8–14].

In 2011, Zeng et al. [15] proposed a wavelet fusion algorithm for visible polarization images, which selected the adaptive weighted fusion rule of window space frequency in high frequency sub-bands and introduced a simulated annealing algorithm. However, the algorithm relied on experience in determining parameters. In 2020, Shen et al. [16] proposed a wavelet based contourlet transform (WBCT) polarization image fusion algorithm together with a fusion rule combining regional characteristic energy and principal component analysis (PCA) transformation. However, this method was very sensitive to noise. Jiang et al. [17] proposed an infrared polarization image fusion algorithm based on nonsubsampled shearlet transform (NSST), decomposed the original image by NSST. They adopted a fusion rule which combined regional correlation degree and regional variance for the low frequency component, and combined regional correlation degree and regional characteristic energy for the high frequency component. The objective evaluation index of this algorithm is better than other algorithms compared in their paper. In 2021, Shi et al. [18] proposed an infrared polarization adaptive selective fusion method based on discrete wavelet transform (DWT). The original image was decomposed by DWT transform, and eight images were obtained by different fusion rules. Then the image with the maximum fuzzy integral was calculated by Choquet fuzzy integral as the best fusion image. The results showed that the fusion algorithm could highlight the common information of infrared polarization and light intensity images while preserving their unique information, and could improve the details of the fusion image. Yang et al. [19] proposed an image fusion algorithm based on two-dimensional discrete wavelet transform, which fused polarization angle images and light intensity images. The fusion image obtained by the algorithm effectively raised the contrast between the target and the background, and enhanced the visual effect of underwater images. In 2022, Wang et al. [20] proposed an infrared polarization image fusion algorithm based on Laplacian pyramid transform. The defect features of photovoltaic cells in the fusion images were more prominent than in traditional image, and evaluations of information entropy and standard difference were significantly improved. Wang and Xu [21] proposed an underwater polarization image fusion algorithm based on Retinex and wavelet transform, which significantly improved the quality of fused images. In 2023, Chen et al. [22] proposed an image fusion method based on multi-scale structure decomposition to achieve fusion of infrared light intensity and polarization image. In their algorithm, the infrared image and polarization map were decomposed into three independent parts: average intensity, signal intensity and signal structure. The decomposition process was replaced by mean filtering, and the final fusion image was obtained by up-sampling and down-sampling. Compared with other algorithms, the algorithm had advantages in four evaluation indices, and subjectively retained more texture details, while also improving contrast and artifact suppression. Meng et al. [23] proposed a color image fusion method, which effectively improved image contrast and retained color information. Gao et al. [24] proposed an adaptive underwater polarization image fusion method, using NSST and simplified pulse coupled neural network, (SPCNN) to process the image, and the detailed contouring and features of underwater objects were highlighted. Yang and Wang [25] proposed a face image enhancement method based on polarization image fusion, which used wavelet packet transform to fuse the facial light intensity image and facial degree of polarization image, and the facial details and contours of the fused image were obvious, with high contrast.

In this paper, we present an image fusion algorithm that combines light intensity and polarization image, by applying on the nonsubsampled contourlet transform (NSCT). The proposed method begins by denoising the original image and then decomposing it using the NSCT transformation. We select the low-frequency sub-band coefficients, with large absolute values, as the fusion coefficients for the low-frequency sub-band. To preserve the edge details of the target effectively, we introduce a novel high-frequency sub-band fusion rule; the high-frequency sub-band coefficients are chosen as the fusion coefficients for the high-frequency sub-band. Subsequently, we reconstruct the fusion image using the NSCT inverse transformation, followed by an assessment of the quality of the fused image. Experimental results demonstrate that, in comparison to the original image and to other fusion algorithms, our proposed algorithm exhibits enhancements according to both subjective and objective evaluation metrics. It excels at retaining edge information from the original image. The fused image can complement polarized information as supplementary light intensity data, and provide a reference for the use of a multi-dimensional optical information feature fusion method for improving the target recognition accuracy in power inspection.

The main contributions of the proposed research article are the following:

1. A denoising method based on noise template threshold matching was employed, effectively addressing the issue of high noise in the polarization degree images.
2. An effective algorithm for the fusion of polarized and intensity images was proposed, utilizing NSCT decomposition to obtain the high and low-frequency components of polarized and intensity images. The algorithm introduced a selection strategy for high-frequency sub-band coefficients based on an edge-preserving operator and a fusion rule for low-frequency sub-bands based on the absolute values of the sub-band coefficients.
3. The experiments in this study indicate that the polarized fusion images obtained using the proposed algorithm not only preserve the visual authenticity of the intensity images but also retain the polarization information from the polarized images. In comparison with other advanced algorithms, the fusion images exhibit better subjective consistency with human visual perception and demonstrate favorable results in objective evaluation metrics.

## Polarization image acquisition

The polarization state of light is often described by Stokes vectors [26, 27]. The four Stokes vectors can be represented in terms of the average of the light intensity in different directions:

1 $$S = \left[ {\begin{array}{*{20}c} {S_{0} } \\ {S_{1} } \\ {S_{2} } \\ {S_{3} } \\ \end{array} } \right] = \left[ {\begin{array}{*{20}c} {I_{{0^{^\circ } }} + I_{{90^{^\circ } }} } \\ {I_{{0^{^\circ } }} - I_{{90^{^\circ } }} } \\ {I_{{45^{^\circ } }} - I_{{135^{^\circ } }} } \\ {I_\text{R} - I_\text{L} } \\ \end{array} } \right],$$

where $S_{0}$ represents the sum of the light intensity values of any two orthogonal polarization directions; $S_{1}$ represents the difference between the polarized light intensity $I_{{^{{0^{^\circ } }} }}$ in the *x*-axis direction and $I_{{^{{90^{^\circ } }} }}$ in the *y*-axis direction; $S_{2}$ represents the difference between the intensity $S_{2}$ of linearly polarized light at 45° from the *x*-axis and the intensity $I_{{^{{135^{^\circ } }} }}$ of linearly polarized light at 135° from the *x*-axis; $S_{3}$ represents the difference between the intensity values of right-handed circularly polarized light $I_{{\text{R}}}$ and left-handed circularly polarized light $I_{{\text{L}}}$.

Degree of polarization, angle of polarization and other parameters are often used to describe the polarization state of light in practical applications.

The degree of linear polarization (DoLP) is defined as the ratio of the intensity of linearly polarized light to the total intensity of light, expressed by Stokes parameters:

2 $${\text{DoLP}} = \frac{{\sqrt {S_{1}^{2} + S_{2}^{2} } }}{{S_{0} }} \times 100\% .$$

In the case of passive imaging, the degree of polarization mainly reflects the polarization capability of the target. Smooth objects and metal objects have a high degree of polarization, while natural backgrounds generally have a low degree of polarization.

Angle of polarization (AoP) is defined as the angle between the direction of the strongest vibration of the electric field vector and the *x*-axis, expressed by Stokes parameters:

3 $${\text{AoP}} = \frac{1}{2}\tan^{ - 1} \frac{{S_{2} }}{{S_{1} }}.$$

**Fig. 1:**
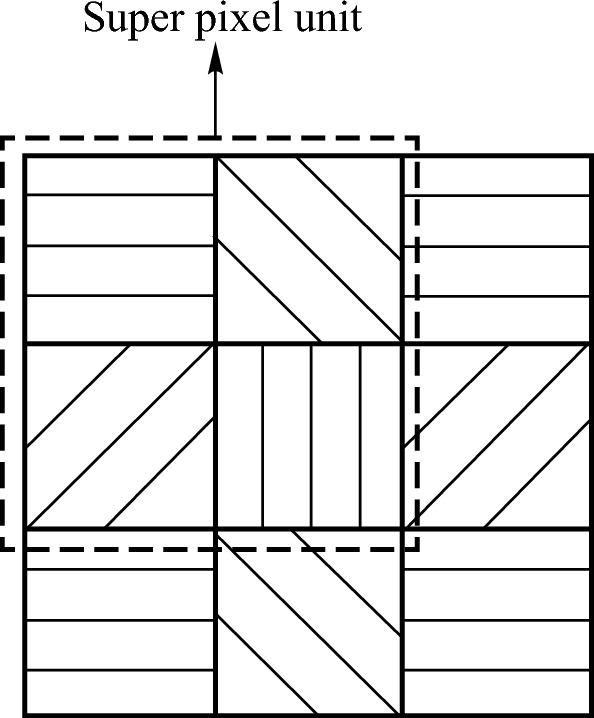
Schematic diagram of division of focal-plane polarimeter of super pixel

The methods for acquiring polarization images include division of time polarimeter (DoTP), division of aperture polarimeter (DoAP), division of amplitude polarimeter (DoAmP), division of focal-plane polarimeter (DoFP), etc. [28–31]. DoFP has the advantages of high efficiency and simultaneously capturing images in four different polarization directions, so this type of polarization camera is selected to obtain polarization-based images in this paper. The principle of Division of Focal Plane Polarimetry imaging involves placing micro linear polarizers in front of the pixels of an image sensor. These polarizers are typically oriented in four directions, such as 0°, 45°, 90°, and 135°. Every group of four pixels, each with a different polarization direction, forms what is known as a super pixel unit. This arrangement allows for the simultaneous capture of light intensity from four distinct polarization directions in a single shot, as shown in Fig. 1.

The polarization camera model used is the Hikvision MV-CH050-10UP, which is equipped with Sony’s IMX250MZR sensor. This camera boasts a resolution of 2448 × 2048 pixels, enabling simultaneous capture of light intensity data at 0°, 45°, 90°, and 135°. Subsequently, utilizing Eqs. (1) through (3), we can compute and construct the polarization image and its associated polarization parameters.

## Polarization image fusion

### Polarization image denoising preprocessing

Both the degree of polarization and the angle of polarization are influenced by noise from the four polarization directions. Using the standard error propagation coefficient method, the noise variances for degree of polarization and angle of polarization can be expressed as [32]:

4 $$\begin{gathered} \sigma_\text{DoLP}^{2} = \left( {\frac{\partial \text{DoLP}}{{\partial I_{{0^{^\circ } }} }}} \right)^{2} \sigma_{{I_{{0^{^\circ } }} }}^{2} + \left( {\frac{\partial \text{DoLP}}{{\partial I_{{45^{^\circ } }} }}} \right)^{2} \sigma_{{I_{{45^{^\circ } }} }}^{2} + \left( {\frac{\partial \text{DoLP}}{{\partial I_{{90^{^\circ } }} }}} \right)^{2} \sigma_{{I_{{90^{^\circ } }} }}^{2} + \left( {\frac{\partial \text{DoLP}}{{\partial I_{{135^{^\circ } }} }}} \right)^{2} \sigma_{{I_{{135^{^\circ } }} }}^{2} , \\ \end{gathered}$$

5 $$\begin{gathered} \sigma_\text{AoP}^{2} = \left( {\frac{\partial \text{AoP}}{{\partial I_{{0^{^\circ } }} }}} \right)^{2} \sigma_{{I_{{0^{^\circ } }} }}^{2} + \left( {\frac{\partial \text{AoP}}{{\partial I_{{45^{^\circ } }} }}} \right)^{2} \sigma_{{I_{{45^{^\circ } }} }}^{2} + \left( {\frac{\partial \text{AoP}}{{\partial I_{{90^{^\circ } }} }}} \right)^{2} \sigma_{{I_{{90^{^\circ } }} }}^{2} + \left( {\frac{\partial \text{AoP}}{{\partial I_{{135^{^\circ } }} }}} \right)^{2} \sigma_{{I_{{135^{^\circ } }} }}^{2} , \\ \end{gathered}$$

where $\sigma_{{I_{{0}^{^\circ} }}}$, $\sigma_{{I_{{45}^{^\circ} }}}$, $\sigma_{{I_{{90}^{^\circ} }}}$, and $\sigma_{{I_{{135}^{^\circ} }}}$ represent the standard deviations of noise in the four polarization directions. The noise variance of each of degree of polarization and angle of polarization is the weighted sum of the noise variance of four polarization directions, so the polarization parameter is very sensitive to noise and the polarization image needs to be de-noised before it is fused with the light intensity image.

Research has demonstrated that noise in degree of polarization images follows a Gaussian distribution [33]. In this context, Miao et al. [34] introduced a polarization image denoising approach based on noise template threshold matching. This method involves identifying the background noise area artificially. It then generates a Gaussian white noise image with the same mean and standard deviation as the background noise region. Subsequently, it evaluates the pixel differences between the polarization image and the generated Gaussian white noise image. Pixels with values less than the pre-defined threshold are set to zero.

When dealing with a substantial volume of images, manual identification of the background noise area is impractical. Moreover, generating a white Gaussian noise image introduces inherent randomness, which can lead to random errors. Furthermore, the assumption that background pixel values are theoretically zero, as suggested by Ref. [31], does not always hold in complex environments. Therefore, this paper presents refinements in three key aspects: the method of identifying the background noise region, the configuration of the noise template, and the setting of background pixel values. The improved denoising algorithm’s specific steps are as follows:

1. The degree of polarization image $g$ is subjected to a grid-based partitioning. Each degree of polarization image, of dimensions 1024 × 1224 pixels, is divided into 64 × 68 small cells, with each cell containing 16 × 18 pixels. The mean and standard deviation of each cell are calculated separately.

6 $$\mu_{ij} = \frac{{\sum\limits_{x = 1}^{x = 16} {\sum\limits_{y = 1}^{y = 18} {g(x,y)} } }}{16 \times 18},$$

7 $$\sigma_{ij} = \sqrt {\frac{{\sum\limits_{x = 1}^{x = 16} {\sum\limits_{y = 1}^{y = 18} {[g(x,y)} } - \mu {}_{ij}]^{2} }}{16 \times 18 - 1}} ,$$

where $g(x,y)$ represents the gray value at the pixel point (*i*, *j*). Because the target makes the standard deviation of the image larger, the part with the smallest standard deviation can be considered to be the background noise region of the image. Note the mean and standard deviation of the background noise region are $\mu$ and $\sigma$, respectively.

2) The absolute value $a$ is obtained by the difference between the pixel value $g(x,y)$ of the original image and the mean value $\mu$ of the background noise region:
  8 $$a = \left| {g(x,y) - \mu } \right|.$$

According to three-sigma rule, if the value of $a$ corresponding to a pixel is found to be less than $3\sigma$, the pixel is considered to be a noise point. The pixel value of the noise pixel is equivalent to the mean value $\mu$ of the background noise region to obtain the noise reduction image $f$.

### Polarization image fusion

In pixel-level image fusion, multi-scale decomposition fusion is the most widely used image fusion algorithm, and is mainly divided into three steps: multi-scale decomposition, sub-band coefficient fusion according to certain rules, and image reconstruction. The selection of image decomposition method and fusion rule is the key to image fusion.

Image decomposition methods include pyramid decomposition, Wavelet transform, Curvelet transform and so on. Wavelet transform has unique advantages in image processing, such as perfect reconstruction capability, low energy loss, and little redundancy. However, the traditional wavelet transform does not have translation invariance, which results in the final fusion image exhibiting blocking artifacts. The Curvelet transform possesses the multi-resolution and time–frequency localization analysis characteristics of discrete Wavelet transform. It also exhibits anisotropy and strong directionality, allowing for a precise and sparse representation of edge information in images with fewer nonzero coefficients, to approximate the curved singular features of the image. However, the Curvelet transform comes with higher computational complexity; it cannot be seamlessly integrated into the multi-resolution analysis framework of images, and, due to the down-sampling operation during execution, it lacks translation invariance, resulting in the occurrence of Pseudo-Gibbs artifacts during reconstruction. The Contourlet transform features multi-scale and multi-directional characteristics, and it can effectively address nearly all the problems that can be solved by the Wavelet transform. The NSCT not only inherits the multi-scale and multi-directional characteristics of the Contourlet transform but also incorporates translation invariance, which results in a more concentrated distribution of coefficient energy after transformation. Consequently, the quality of image fusion is enhanced. NSCT not only has the characteristics of multi-scale and multi-direction, but also has translation invariance, which makes the coefficient energy after transformation more concentrated, so the image fusion quality is improved. The support interval of the NSCT transform takes the form of a rectangular structure with variable scale, significantly reducing the number of elements required to approximate image edges at different resolutions compared to the Wavelet transform requirement. Consequently, NSCT transform excels at representing the edge characteristics of an image, leading to a more concentrated energy distribution in the coefficient representation, ultimately facilitating sparse representation of curves.

**Fig. 2:**
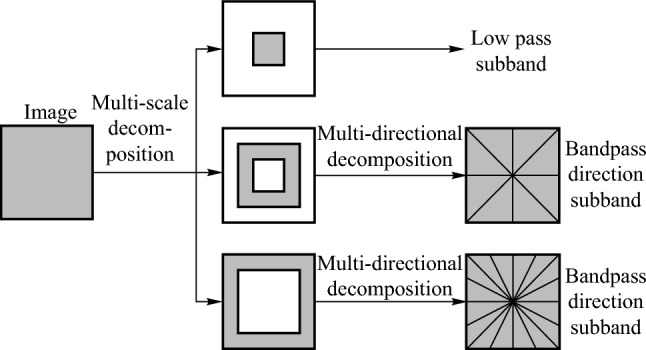
NSCT structure diagram

The NSCT primarily comprises two components: multi-scale decomposition and multi-directional decomposition. The multi-scale decomposition is carried out using the nonsubsampled pyramid (NSP), while the multi-directional decomposition is achieved through the nonsubsampled directional filter bank (NSDFB). The structure of the NSCT is illustrated in Fig. 2.

In the actual application process, the decomposition can be adjusted according to the need to obtain the desired information. In view of the many advantages of NSCT transform, this paper chooses NSCT transform as the image decomposition method.

**Fig. 3:**
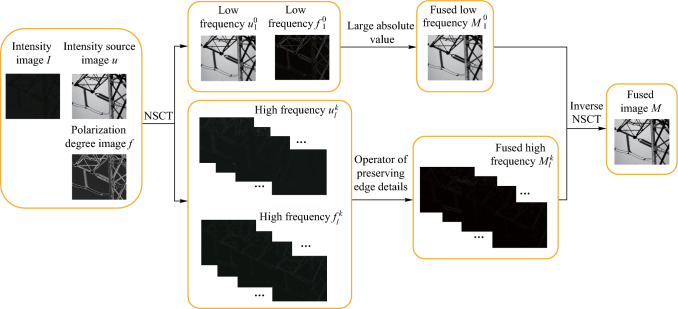
Flow chart of image fusion algorithm

In the selection of fusion rules, the absolute maximum principle is adopted in the low frequency sub-band to preserve the energy of the low frequency sub-band as much as possible. The operator of preserving edge details is chosen in the high frequency sub-band to preserve the prominent edge and texture details of the polarization image. The fusion algorithm process is shown in Fig. 3.

The specific steps of the algorithm are as follows:

1. First, the source image $u$ is obtained by histogram equalization of the light intensity image, so as to improve the contrast of the light intensity image under low illumination.
2. Perform NSCT decomposition on the registered source image $f$ and $u$ to decompose four layers, then obtain the corresponding low-frequency sub-band image $f_{1}^{0}$, $u_{1}^{0}$, and the corresponding high-frequency sub-band, $f_{l}^{k}$, $u_{l}^{k}$, where $l = 2,3,4$, $k = 1,2,...,2^{l - 1}$; *l* represents the number of decomposition layers and *k* represents the number of high-frequency sub-bands decomposed in the direction of *l* layer.
3. Low-frequency sub-band fusion: the low-frequency sub-band mainly contains most of the energy and a few details of the original image, where the energy is defined as the sum of the squares of pixel intensities within the image. To retain the energy of the low-frequency sub-band, the low-frequency sub-band coefficient with large absolute value is selected as the low-frequency sub-band fusion coefficient $M_{1}^{0}$.
4. High-frequency sub-band fusion: the high-frequency sub-band mainly contains the edge and texture details of the image. To retain the edge and texture details of the image, the high-frequency sub-band coefficient with strong edge retention ability is selected, and the edge retention ability is measured by Eq. (9):
  9 $$\begin{aligned} {\text{NAM}}_{2} =  & (w_{1} * I_{{\text{h}}} )^{2} + (w_{2} * I_{{\text{h}}} )^{2} \\ +   & (w_{3} * I_{{\text{h}}} )^{2} + (w_{4} * I_{{\text{h}}} )^{2} , \end{aligned}$$

while

$$w_{1} = \left[ {\begin{array}{*{20}c} { - 1} & { - 1} & { - 1} \\ 2 & 2 & 2 \\ { - 1} & { - 1} & { - 1} \\ \end{array} } \right]$$, $$w_{2} = \left[ {\begin{array}{*{20}c} { - 1} & 2 & { - 1} \\ { - 1} & 2 & { - 1} \\ { - 1} & 2 & { - 1} \\ \end{array} } \right]$$, $$w_{3} = \left[ {\begin{array}{*{20}c} { - 1} & 0 & 0 \\ 0 & 2 & 0 \\ 0 & 0 & { - 1} \\ \end{array} } \right]$$, $$w_{4} = \left[ {\begin{array}{*{20}c} 0 & 0 & { - 1} \\ 0 & 2 & 0 \\ { - 1} & 0 & 0 \\ \end{array} } \right]$$, where * represents the convolution operation, $w_{1}$, $w_{2}$, $w_{3}$, and $w_{4}$ represent four operators that preserve edge details, and $I_{{\text{h}}}$ represents the high frequency sub-band coefficients. Similarly, the high-frequency sub-band coefficient with large ${\text{NAM}}_{2}$ coefficient is selected as the high-frequency sub-band fusion coefficient $M_{l}^{k}$ based on the comparison of the ${\text{NAM}}_{2}$ coefficient calculated by Eq. (9), where *l* represents the number of decomposition layers and *k* represents the number of high-frequency sub-bands decomposed in the direction of *l* layer.

5) The fusion image can be obtained by inverse NSCT transformation of the low frequency and high frequency sub-band coefficients after fusion.

## Analysis of experimental results

### Evaluation function

To evaluate the experimental results, it is necessary to evaluate the image quality objectively. The objective evaluation indices of image quality are divided into reference image quality evaluation and non-reference image quality evaluation. Reference image quality evaluation requires a real reference image to measure the similarity between the processed image and the real image, which is often used in image denoising, image compression and other fields. Commonly used reference image quality evaluation indexes include mean squared error (MSE), peak signal-to-noise ratio (PSNR), and structural similarity (SSIM). Non-reference image quality evaluation is applicable to the case without reference image, and is often used in image fusion and other fields. Therefore, three non-reference image quality evaluation indexes, namely information entropy (IE), standard deviation (SD) and average gradient (AG), are selected in this paper to evaluate the fusion results of each algorithm.

The IE is a metric for assessing the richness of information in an image. A higher IE value indicates a wider grayscale distribution, typically corresponding to a greater amount of information in the image. IE is defined as follows:

10 $${\text{IE}} = - \sum\limits_{i = 0}^{L} {P(l)} \log_{2} P(l),$$

where *l* is the total gray level of the image, and $P(l)$ is the proportion of the number of pixels with gray value l to the total number of pixels in the image.

The SD reflects the degree of dispersion of the grayscale relative to the average grayscale. A higher SD in an image corresponds to a greater dispersion of grayscale values. Generally, this corresponds to a richer texture detail in the image. SD is defined as follows:

11 $${\text{SD}} = \sqrt {\frac{1}{M \times N}\sum\limits_{i = 1}^{M} {\sum\limits_{j = 1}^{N} {\left[ {f(i,j) - u} \right]} }^{2} } ,$$

12 $$u = \frac{1}{M \times N}\sum\limits_{i = 1}^{M} {\sum\limits_{j = 1}^{N} {f(i,j)} } ,$$

where *M* and *N* are the rows and columns of the image, $f(i,j)$ represents the gray value of the image at the point (*i*,*j*), and *u* represents the average gray value of the image.

The AG reflects the variations in detail and texture within the image, signifying the level of clarity of an image. A greater AG signifies more pronounced texture features within the image, indicative of enhanced detail transformation capabilities. AG is defined as follows:

13 $${\text{AG}} = \frac{{\sum\limits_{i = 1}^{M} {\sum\limits_{j = 1}^{N} {\left[ {(\Delta_{i} f(i,j))^{2} - (\Delta_{j} f(i,j))^{2} } \right]} }^{\frac{1}{2}} }}{M \times N},$$

where *M* and *N* are the rows and columns of the image, and $\Delta_{i} f(i,j)$ and $\Delta_{j} f(i,j)$ represent the gray value gradients on the rows and columns of the image, respectively.

### Experimental results and analysis

**Fig. 4:**
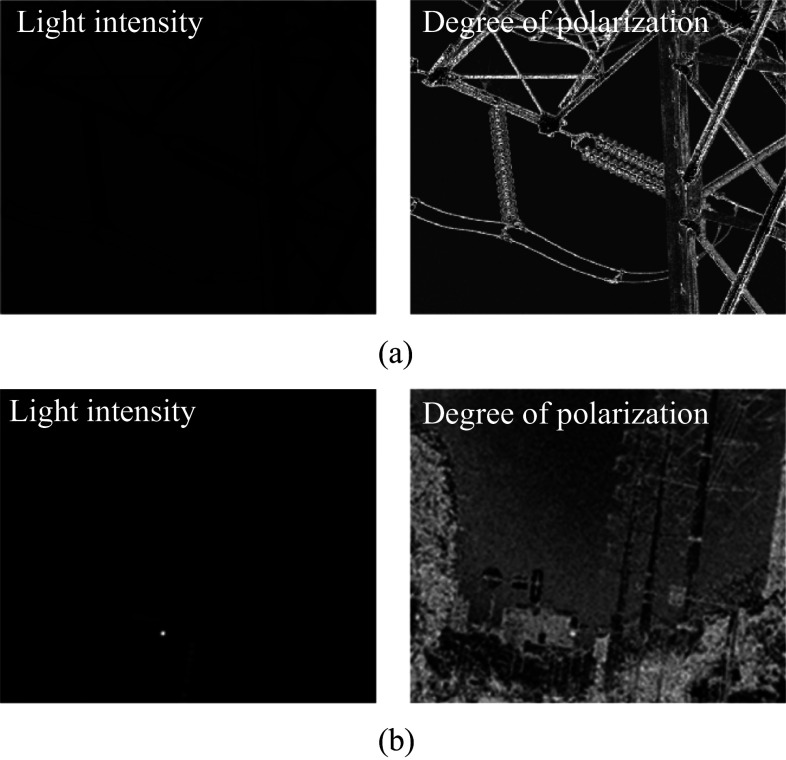
Intensity image and polarization image under different scenes. **a** Scene 1. **b** Scene 2

In this paper, two distinct scenes were selected for experimental investigation. To demonstrate the advantages of polarization imaging in highlighting target edges and the efficacy of the denoising algorithm presented in this paper, low-light conditions were chosen for image capture. The first scene involved a close-up view of a high-voltage power tower, while the second scene featured a distant view of the same tower. The intensity and polarization images for both scenes are illustrated in Fig. 4. All images presented in this paper were captured on-site by the authors.

**Fig. 5:**
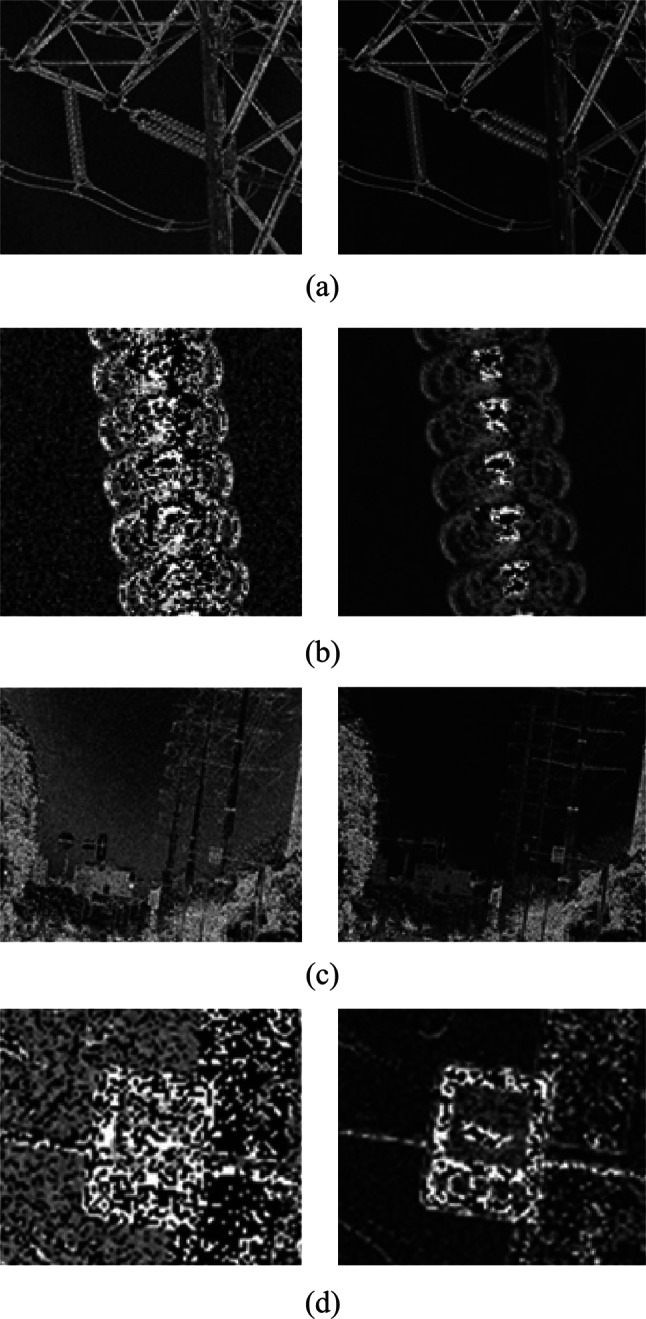
Polarization images of different scenes before and after noise reduction. **a** Polarization image of Scene 1 before and after denoising. **b** Zoom-in details of the insulator in Scene 1 before and after denoising. **c** Polarization image of Scene 2 before and after denoising. **d** Zoom-in details of the street signs in Scene 2 before and after denoising

As shown in Fig. 4, under low-light conditions, the target is difficult to discern in the light intensity image, while the polarization image clearly reveals the target. This is attributed to the inherent property of polarization information, which remains unaffected by variations in light intensity. However, in low-light environments, polarization images tend to exhibit a low signal-to-noise ratio and high noise levels. Therefore, as outlined in Sect. 2.1, a denoising method is employed for preprocessing the polarization images. The denoised polarization images, before and after preprocessing, are illustrated in Fig. 5 (the left image represents the image prior to denoising, and the right image represents the image post-denoising).

From Fig. 5, it is evident that the denoising algorithm exhibits favorable noise suppression in both the background and target regions of the degree of polarization image. The SNR of the Scene 1 image has been increased from 0.4505 to 0.5387. The SNR of the Scene 2 image has been increased from 0.4948 to 0.5524.

The polarization image after denoising processing is fused with the light intensity image. To verify the effectiveness of the fusion algorithm in this paper, nine different types of multi-scale image fusion algorithms are selected to compare with the fusion algorithm proposed in this paper. The different fusion algorithms are shown in Table 1.

**Table 1:** Different image fusion algorithms

Method	Method of decomposition	Method of fusion
Method 1 [21]	Single wavelet transform	Low frequency: saliency map High frequency: maximization of absolute value
Method 2 [35]	Multiwavelet transform	Low frequency: mean value High frequency: maximum energy
Method 3 [35]	Curvelet transform	Low frequency: mean value High frequency: maximum energy
Method 4	Nonsubsampled shearlet transform	Method in this paper
Method 5 [20]	Laplacian pyramid	Low frequency: regional energy weighted average High frequency: regional energy maximum
Method 6 [22]	Multiscale structure decomposition	Low frequency: weighted average High frequency: power function weighted average
Method 7 [36]	Nonsubsampled shearlet transform	Low frequency: energy attribute (EA) High frequency: PCNN
Method 8 [37]	Nonsubsampled contourlet transform	Low frequency: PCA-based dictionary learning High frequency: MAX-SML
Method 9 [38]	Nonsubsampled contourlet transform	Low frequency: a local Laplacian energy based fusion rule High frequency: a phase-congruency based fusion rule
Method 10	Nonsubsampled contourlet transform	Method in this paper

**Fig. 6:**
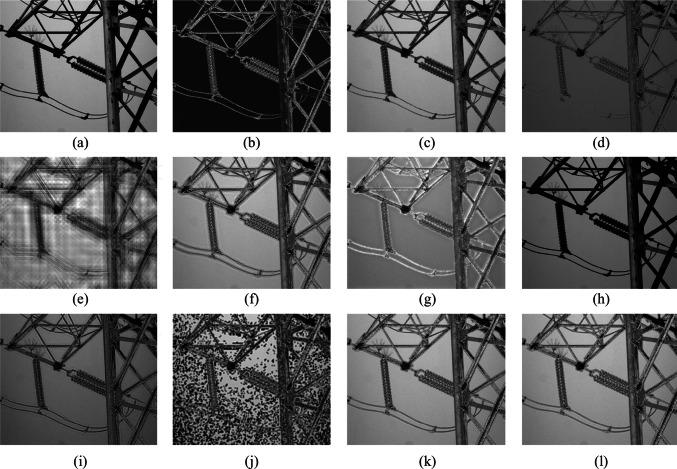
Scene 1 Fusion experiment results of different fusion rules. **a** Processed light intensity image. **b** Processed polarization image. **c**–**l** Fusion images generated by fusion rules methods 1–10, respectively

**Fig. 7:**
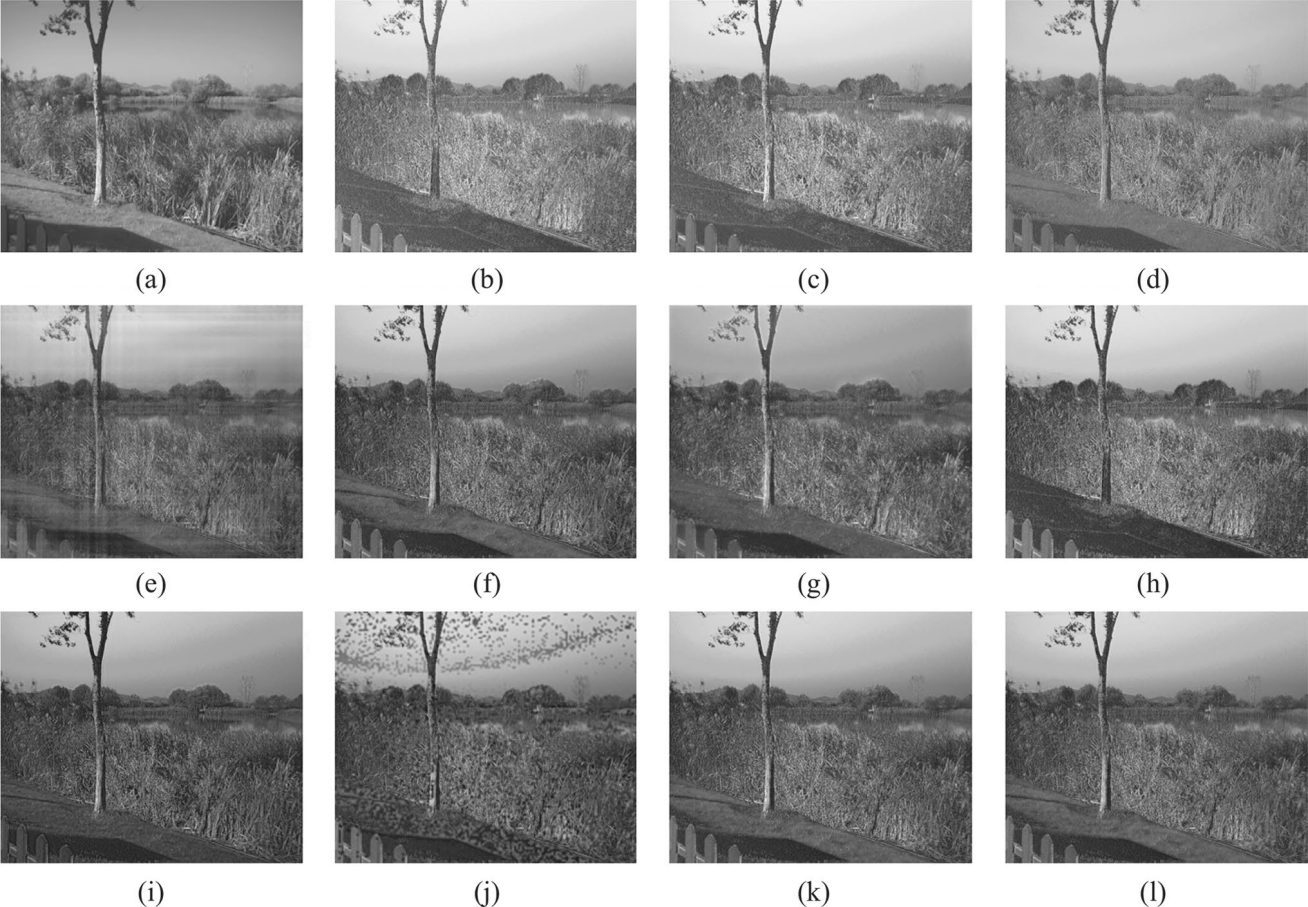
Scene 2 Fusion experiment results of different fusion rules. **a** Processed light intensity image. **b** Processed polarization image. **c–l** Fusion images generated by fusion rules methods 1–10, respectively

**Fig. 8:**
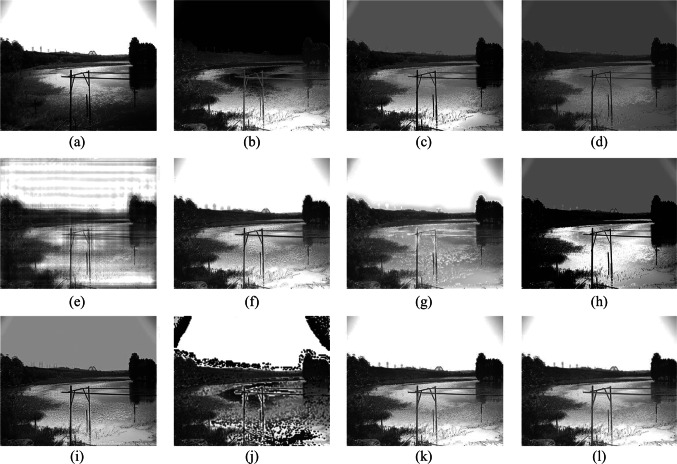
Scene 3 Fusion experiment results of different fusion rules. **a** Processed light intensity image. **b** Processed polarization image. **c**–**l** Fusion images generated by fusion rules methods 1–10, respectively

**Fig. 9:**
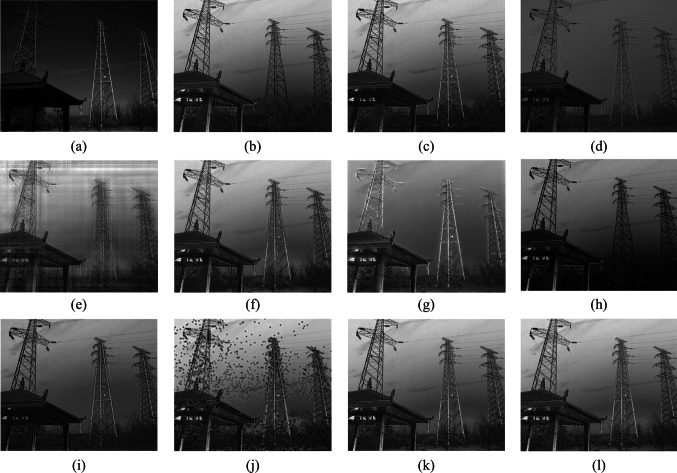
Scene 4 Fusion experiment results of different fusion rules. **a** Processed light intensity image. **b** Processed polarization image. **c**–**l** Fusion images generated by fusion rules methods 1–10, respectively

**Fig. 10:**
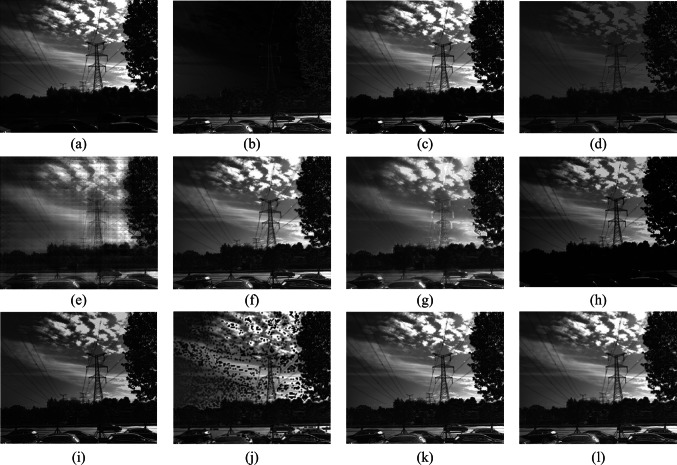
Scene 5 Fusion experiment results of different fusion rules. **a** Processed light intensity image. **b** Processed polarization image. **c**–**l** Fusion images generated by fusion rules methods 1–10, respectively

To confirm the effectiveness of the fusion algorithm in this paper, images of five different scenes are taken, covering targets such as towers, lakes, and cars. Scene 1 is an image of an insulator in low illumination; Scenes 2 and 3 are images of a lake surface; Scene 4 is an image of a tower and a pavilion; Scene 5 is an image of cars and road surface. The fusion results for each scene using methods 1 to 10 are illustrated in Figs. 6, 7, 8, 9, and 10. In these figures, (a) represents the intensity image, (b) is the polarization image, and (c) to (l) correspond to methods 1 to 10, respectively.

In Scene 1, the polarization of degree images effectively emphasizes the edge contour information of insulators. However, three images, (d), (h), and (i), exhibit an overall darkening, with (d) experiencing partial information loss. The image quality of (e) and (j) significantly deteriorates, displaying severe distortion. In images (g) and (f), excessive emphasis on polarimetric information leads to a deviation from human visual perception. Partial loss of polarimetric characteristics is observed in image (c). Conversely, images (k) and (l) successfully preserve the polarimetric characteristics of the insulators.

In Scene 2, the fence in the bottom-left corner is difficult to discern in the intensity image due to shadows but is highlighted in the polarization of degree image. Images (e) and (j) exhibit noticeable distortion. The polarimetric characteristics of the fence in images (c), (d), and (i) are not prominently emphasized. The intensity information of the grass is lost in images (c) and (h). Artifacts appear around the mountains in image (g). The fusion results in images (f), (k), and (l) are comparatively favorable, effectively highlighting the fence in the shadows.

In Scene 3, the stone steps in the bottom-left corner are difficult to discern in the intensity image, but are easily visible in the polarization of degree image. Additionally, the polarization of degree image effectively eliminates the reflections on the lake surface. The power poles in the distance are difficult to distinguish in images (c), (d), and (g). In image (h), the steps are obscured, polarimetric information is lost, and the image exhibits high black-and-white contrast, impeding observation. Images (e) and (j) suffer from significant distortion. Notably, image (f) exhibits pronounced artifacts around the distant power poles. Fusion results in image (i), (k), and (l) effectively combining intensity and polarization information, with minimal artifacts and suitable contrast.

In Scene 4, the polarization of degree images effectively highlights the texture details inside the pavilion and the insulators on the power poles. Severe distortion is observed in images (e) and (j). Images (d), (g), and (i) lose the intensity information of clouds in the sky. Numerous artifacts appear at the edges of the power poles in image (f). The foliage beneath the poles is too dark in images (c) and (h), resulting in the loss of many details in the intensity image. The contours in images (k) and (l) are sharp, with minimal artifacts, effectively preserving both intensity and polarization information.

In Scene 5, the road surface and vehicles show a high degree of polarization, making them prominent in the polarization of degree images. Severe distortion is observed in images (e) and (j). Images (d) and (h) experience significant loss of polarization information for the road surface and vehicles. Image (c), the texture details of vehicles are not sufficiently clear. Image (g) exhibits pronounced artifacts around the power poles and foliage, resulting in reduced image clarity. In image (i), the polarization information for the car windows is not distinctly expressed. Images (f), (k), and (l) are clear, preserving both intensity and polarization information.

For objective evaluation, we apply three evaluation metrics, IE, SD, and AG, to assess the fusion results of each algorithm. Tables 2, 3, 4, 5, and 6 present the objective evaluation metrics for various fusion rules in the five different scenes, respectively.

**Table 2:** Comparison of objective evaluation indexes of Scene 1 fusion experiment

Methods	IE↑	SD↑	AG↑
1	7.0678	66.9075	161.3175
2	6.5347	49.0444	108.1702
3	7.4030	63.2892	158.4594
4	7.0677	67.4791	168.3071
5	7.0819	43.2988	166.9415
6	6.1726	65.9628	110.6997
7	6.7405	55.2197	124.2322
8	7.8167	65.8935	126.7104
9	6.7272	68.2897	169.9893
10	6.8238	67.9096	169.9650

**Table 3:** Comparison of objective evaluation indexes of Scene 2 fusion experiment

Methods	IE↑	SD↑	AG↑
1	7.4892	60.6004	89.2141
2	7.2724	47.3143	77.4960
3	7.5508	55.5094	96.3626
4	7.3183	57.9388	97.7732
5	7.2552	43.2697	95.4753
6	7.3209	58.9307	77.4642
7	7.3261	55.3230	82.1570
8	7.4838	52.1646	88.0415
9	7.2238	57.8648	98.1550
10	7.2723	57.6674	98.1523

**Table 4:** Comparison of objective evaluation indexes of Scene 3 fusion experiment

Methods	IE↑	SD↑	AG↑
1	6.0925	62.4756	139.1531
2	5.9369	44.2863	119.3052
3	6.8284	72.7687	186.3628
4	5.8291	77.6301	189.6079
5	6.2976	70.4839	189.5631
6	4.8481	84.5428	129.0526
7	6.2160	68.3044	157.0929
8	6.3984	85.1588	156.1318
9	5.7261	77.4021	190.2017
10	5.7570	77.1638	190.1982

**Table 5:** Comparison of objective evaluation indexes of Scene 4 fusion experiment

Methods	IE↑	SD↑	AG↑
1	7.8263	68.3847	138.1880
2	7.0426	40.0483	101.9926
3	7.8015	60.8645	142.9512
4	7.7607	63.6687	144.2017
5	7.5265	48.3939	137.8227
6	7.5142	66.4720	102.5889
7	7.6701	58.4805	119.5504
8	7.7728	63.7692	134.4087
9	7.6602	63.6789	144.6936
10	7.7161	63.3082	144.6926

**Table 6:** Comparison of objective evaluation indexes of Scene 5 fusion experiment

Methods	IE↑	SD↑	AG↑
1	7.7698	68.1424	115.9455
2	7.0829	37.7573	73.4206
3	7.8613	62.8570	114.2830
4	7.8567	67.8635	115.4858
5	7.7628	62.6318	117.1826
6	6.2116	72.8339	76.4506
7	7.6966	63.8216	92.8949
8	7.7614	64.5921	100.9932
9	7.8078	68.2158	115.6551
10	7.8298	67.9535	115.6372

The first, second, and third highest values in the table are highlighted in boldface, italics and underline. Among the three objective evaluation metrics, our algorithm demonstrates superiority in the SD and AG metrics, while in the IE metric it does not exhibit superiority compared to other methods. Methods 3 and 8 perform best in terms of the IE metric; however, the fused images obtained from these methods exhibit severe image quality degradation, introducing a significant amount of irrelevant information. Such irrelevant information leads to the dispersion of the gray level, which initially represented the target in the image, across other gray level that are irrelevant to the image’s content, resulting in *P*(*l*) in Eq. (10) becoming smaller, and thus increasing the information entropy of the image. Consequently, the IE metric is highest for these methods. Excluding these two methods, our algorithm also performs well in terms of the IE metric. Various methods exhibit different performance in terms of SD on different images. Among them, methods 1, 4, 6, 9, and our proposed method show relatively good performance. However, methods 1 and 6 lack sufficient emphasis on polarization features, resulting in an overly prominent black-and-white contrast that compromises the ability to represent details. Method 4 exhibits a significant presence of artifacts. On the other hand, methods 9 and the method proposed in this paper perform well in terms of SD, delivering good image quality. Methods 9 and the proposed method exhibit high AG across various scenes, indicating that the fusion rules designed in our algorithm outperform the those of other algorithms in terms of edge and detail preservation. Multiscale fusion, being one of the most widely applied techniques in the field of image fusion, offers the advantage of adaptability to different application scenes through the design of specific fusion rules. The fusion rules devised in this paper are tailored to emphasize target edges, making them suitable for object detection tasks. Both subjective and objective evaluation metrics indicate that the designed rules effectively achieve their intended purpose.

## Conclusion

To use the polarization information as a supplement to the light intensity information and better identify the inspection target, this paper carries out denoising pre-processing on the collected polarization images. It designs a multidimensional optical information fusion algorithm based on NSCT transform for light intensity images and polarization images, and proposes a high-frequency fusion rule that selects high-frequency sub-bands according to the edge holding ability, which makes the fusion image better retain the details and edge information of the original image. Experiments are carried out by imaging some common scenes in power grid inspection to validate the algorithm. The results show that the improved algorithm is superior in both subjective vision and objective evaluation indexes. Using this fusion algorithm, the polarization information and light intensity information can be better combined and the advantages of the two kinds of image information can be preserved. Furthermore, this algorithm is applicable to the fusion of registered infrared intensity images and polarization of degree images, as well as the fusion of infrared and visible light images.

The denoising algorithm proposed in this paper is only suitable for removing noise in polarization of degree images with simple backgrounds. Additionally, natural objects with low polarization of degree, such as foliage, can deteriorate the quality of polarization images, leading to a reduction in the quality of the fused images. The fusion algorithm is time-consuming, which is not conducive to practical applications in real-world scenarios. The fusion results in this paper relying on manually selected fusion rules. While these rules can be customized flexibly according to the scene, they lack general applicability. Future directions of this work include designing a denoising algorithm universally applicable to polarization of degree images to enhance the quality of fused images. Specific fusion rules will be devised to address targets with low polarization of degree, reducing their impact on the quality of the fused images. Optimization of the fusion algorithm for faster processing speed will be explored. Research into image fusion algorithms based on deep learning will also be conducted to expand their applicability to different scenarios.

This research work provides a reference for the application of multi-dimensional optical information fusion in power grid inspection, and is expected to improve the accuracy of power inspection and the adaptability to the environment in the subsequent target recognition.

## Declarations

### Availability of data and materials

The data that support the findings of this study are available from the corresponding author, upon reasonable request.
